# Machine learning identifies multi-parametric functional PET/MR imaging cluster to predict radiation resistance in preclinical head and neck cancer models

**DOI:** 10.1007/s00259-023-06254-9

**Published:** 2023-05-06

**Authors:** Simon Boeke, René M. Winter, Sara Leibfarth, Marcel A. Krueger, Gregory Bowden, Jonathan Cotton, Bernd J. Pichler, Daniel Zips, Daniela Thorwarth

**Affiliations:** 1 Department of Radiation Oncology, University of Tübingen, Tübingen, Germany; 2 German Cancer Consortium (DKTK), partner site Tübingen, and German Cancer Research Center (DKFZ), Heidelberg, Germany; 3 Section for Biomedical Physics, Department of Radiation Oncology, University of Tübingen, Hoppe-Seyler-Str. 3, 72076 Tübingen, Germany; 4 Werner Siemens Imaging Center, Department of Preclinical Imaging and Radiopharmacy, University of Tübingen, Tübingen, Germany; 5 Cluster of Excellence iFIT (EXC 2180) “Image Guided and Functionally Instructed Tumor Therapies”, University of Tübingen, Tübingen, Germany

**Keywords:** Radiotherapy, Multi-parametric functional imaging, PET/MRI, Personalized radiation oncology, Dose painting, Machine learning

## Abstract

**Purpose:**

Tumor hypoxia and other microenvironmental factors are key determinants of treatment resistance. Hypoxia positron emission tomography (PET) and functional magnetic resonance imaging (MRI) are established prognostic imaging modalities to identify radiation resistance in head-and-neck cancer (HNC). The aim of this preclinical study was to develop a multi-parametric imaging parameter specifically for focal radiotherapy (RT) dose escalation using HNC xenografts of different radiation sensitivities.

**Methods:**

A total of eight human HNC xenograft models were implanted into 68 immunodeficient mice. Combined PET/MRI using dynamic [18F]-fluoromisonidazole (FMISO) hypoxia PET, diffusion-weighted (DW), and dynamic contrast-enhanced MRI was carried out before and after fractionated RT (10 × 2 Gy). Imaging data were analyzed on voxel-basis using principal component (PC) analysis for dynamic data and apparent diffusion coefficients (ADCs) for DW-MRI. A data- and hypothesis-driven machine learning model was trained to identify clusters of high-risk subvolumes (HRSs) from multi-dimensional (1-5D) pre-clinical imaging data before and after RT. The stratification potential of each 1D to 5D model with respect to radiation sensitivity was evaluated using Cohen’s *d*-score and compared to classical features such as mean/peak/maximum standardized uptake values (SUV_mean/peak/max_) and tumor-to-muscle-ratios (TMR_peak/max_) as well as minimum/valley/maximum/mean ADC.

**Results:**

Complete 5D imaging data were available for 42 animals. The final preclinical model for HRS identification at baseline yielding the highest stratification potential was defined in 3D imaging space based on ADC and two FMISO PCs ($p<0.001$). In 1D imaging space, only clusters of ADC revealed significant stratification potential ($p=0.002$). Among all classical features, only ADC_valley_ showed significant correlation to radiation resistance ($p=0.006$). After 2 weeks of RT, FMISO_c1 showed significant correlation to radiation resistance ($p=0.04$).

**Conclusion:**

A quantitative imaging metric was described in a preclinical study indicating that radiation-resistant subvolumes in HNC may be detected by clusters of ADC and FMISO using combined PET/MRI which are potential targets for future functional image-guided RT dose-painting approaches and require clinical validation.

**Supplementary Information:**

The online version contains supplementary material available at 10.1007/s00259-023-06254-9.

## Introduction

About 50% of patients treated with radiochemotherapy (RCT) for locally advanced human papilloma virus–negative head-and-neck cancer (HNC) experience local and regional treatment failure [1, 2]. As salvage treatment options are limited, locoregional failure in most patients leads to severe symptoms and ultimately to death. Thus, overcoming treatment resistance by optimized RCT represents an important area of research. Preclinical and clinical data demonstrates that tumor hypoxia and other microenvironmental factors significantly contribute to tumor radiation resistance [3–6]. Different quantitative imaging biomarkers (QIBs) related to tumor hypoxia and microenvironment have shown potential for outcome prediction, early response assessment, and RT personalization, e.g., by means of risk adapted radiation dose modulation [7–12].

Hypoxia imaging using positron emission tomography (PET) with specific radiotracers such as [18F]-Fluoromisonidazole (FMISO) has proven prognostic power to predict outcome after RCT in HNC [7, 13–15]. Similarly, functional magnetic resonance imaging (MRI) techniques, such as diffusion-weighted (DW) imaging assessing tumor cellularity or dynamic contrast-enhanced (DCE) imaging which allows to analyze tissue vascularity and vessel permeability, have been correlated to tumor response after RCT in HNC and other solid tumors [8, 9, 16, 17]. Some studies correlated the spatial distribution of multiple QIB and suggested complementary biological information [18–20]. However, the optimal QIB or imaging profile using multiple QIB to predict outcome after RCT in HNC is unknown. Most results were derived from small observational clinical cohorts and none of the previous studies was able to relate relevant QIB to radiation resistance on a biological or pre-clinical level.

Future clinical use of QIB to personalize radiation dose to overcome treatment resistance requires a widely available, robust, affordable, and simple method to generate QIB to allow multicenter trials and easy access for patients. In contrast to molecular profiling [21, 22], liquid biopsy [23, 24], histopathology [25, 26], or combination with immunotherapy [27, 28], QIBs have the benefit of spatial tumor characterization [29] and thus optimal conditions for focal personalized interventions such as dose-painting, including dose escalation and dose de-escalation [13, 30, 31].

The aim of this preclinical study was to develop and train a multi-scale model from a broad and unbiased basis for prediction of high-risk subvolumes (HRS) in HNC linked to increased radiation resistance derived from hypoxia PET, DW-, and DCE-MRI. Multi-parametric small animal PET/MRI of xenograft tumors from different human HNC cell lines with variable, known radiation sensitivities were imaged and evaluated by novel machine learning (ML) methods to identify HRS in multi-dimensional imaging space. The hypothesis to be investigated in this study was therefore that with novel ML approaches new QIB or imaging profiles will be discovered to define HRS in a pre-clinical scenario, which may be used for future personalized radiotherapy (RT) interventions in a clinical setting.

## Material and methods

### Study design, animals, and tumor models

A total of 68 mice with implanted human HNC cell lines of different, known radiation sensitivities were examined with simultaneous functional PET/MRI before and after 2 weeks of fractionated RT. Details on animals, implanted cell lines, imaging data, and time points are summarized in Table 1. The animal facilities and all experiments were approved according to our institutional guidelines and the German animal welfare regulations (animal allowance no. 35/9185.81-2/R4/16). Two to 5 days before tumor cell injection, 4- to 6-week-old immunodeficient female nude mice (NMRI nu/nu, Charles River Laboratories) received a 4-Gy total body irradiation (6 MV photons, Elekta SL15, Crawley, UK) to further suppress the residual immune system. Eight well-established human HNSCC tumor cell lines (UTSCC-45, XF354, UTSCC-14, UTSCC-8, FaDu, UTSCC-5, CAL-33, SAS) with known radiation sensitivities in vivo [32, 33] were grown in cell culture (cf. Table 1). Exponentially growing cells of the third passage were trypsinised, and a single cell suspension with approx. 500,000 cells dissolved in 50 μl phosphate-buffered saline was prepared and injected subcutaneously on the right hind leg of the animal. Animals were checked regularly for weight loss, abnormal behavior, or other signs of distress. Tumor diameter was measured twice weekly. After reaching the target size of 7–10-mm diameter, tumors were examined using multi-modal, small animal PET/MRI before and after 2 weeks of fractionated RT.

**Table 1:** Preclinical data. Details on animals, head-and-neck cancer cell lines including mean and 95% confidence interval (CI) tumor control dose 50% (TCD50) according to [33], radiation sensitivities grouped into high (H), medium (M), medium/low (ML), and low (L) as well as number of complete imaging data sets, data sets with hypoxia positron emission tomography (PET), diffusion-weighted MR imaging (DWI), and dynamic contrast enhanced (DCE) MRI before the start of radiotherapy (RT) and after 14 days

Cell line	TCD50/Gy mean [95% CI]	Radio-sensi-tivity group	Volume/mm^3^ median [range]	# implanted xeno-grafts	# complete imaging data sets before start of RT (PET/DWI/DCE)	# complete imaging data sets after 14 days RT (PET/DWI/DCE)
UTSCC-45	45 [38; 52]	H	895 [721; 1322]	12	8 (9/10/9)	4 (6/6/5)
XF354	47 [40; 55]	H	901 [591; 1091]	4	4 (4/4/4)	4 (4/4/4)
UTSCC-14	52 [46; 59]	H	464 [234; 533]	4	4 (4/4/4)	4 (4/4/4)
UTSCC-8	52 [40; 61]	H	558 [352; 764]	4	2 (3/4/2)	0 (0/0/0)
FaDu	85 [77; 96]	M	1425 [187; 1940]	16	12 (12/12/12)	9 (11/12/10)
CAL-33	105 [90; 141]	ML	322 [262; 665]	8	5 (5/6/5)	3 (5/5/4)
UTSCC-5	117 [103; 140]	L	44 [0; 113]	4	0 (0/0/0)	0 (0/0/0)
SAS	127 [114; 140]	L	1191 [683; 1762]	16	7 (9/11/8)	8 (9/10/8)
Total ${C}_{{a}{{l}}{{l}}}\boldsymbol{ }({{{C}}}_{{{m}}{{a}}{{x}}})$				68	42 (46/51/44)	32 (39/41/35)

### Multi-modal imaging and radiotherapy

**Fig. 1:**
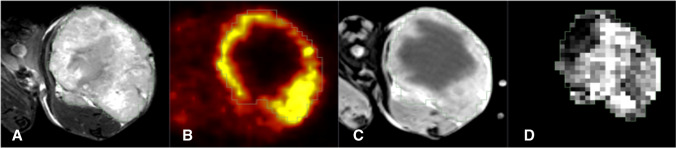
Multi-dimensional pre-clinical imaging data. Example of pre-clinical imaging data consisting of **A** anatomical T2-weighted MRI, **B** FMISO PET, and **C** contrast-enhanced T1-weighted MRI, **D** apparent diffusion coefficients (ADC) derived from diffusion-weighted (DW) MRI

All animals were imaged with combined PET/MRI using a small animal 7-T MRI system with a dedicated PET insert [29, 34, 35]. Animals were anesthetized with a mixture of isoflurane (1.5–2.0%; Abbott, Wiesbaden, Germany) and air (flow rate 1.0–1.5 l/min) with continuous monitoring of the breathing rate and were placed on a warming pad to maintain constant body temperature during imaging. The imaging protocol consisted of simultaneous dynamic FMISO PET, anatomical T2-weighted MRI (T2w-MRI), DW-MRI, and DCE-MRI, with T2w- and DW-MRI in a gated acquisition technique with respiratory triggering (cf. Fig. 1).

Dynamic PET was acquired in listmode for 90 min post injection (p.i.) of approximately 10 MBq FMISO in 200 μl of physiological sodium chloride solution (0.9%) into the animal’s tail vein. PET data was reconstructed to a total of 65 time frames (36 × 10 s, 18 × 60 s, 11 × 360 s) using 2D-OSEM (4 iterations, 8 subsets). DW-MRI was performed with an echo planar imaging sequence with nine equidistant *b*-values (*b* = 0–800 s/mm^2^). DCE-MRI was acquired for a total duration of 13.5 min starting 1 min before injection of the contrast agent (Gadovist®, Bayer Vital GmbH, Germany), with a temporal resolution of 5.4 s. Details about the pre-clinical image acquisition protocol are given in Table 2.

**Table 2:** Details of the pre-clinical PET/MR imaging protocol

	dyn. FMISO PET	T2w MRI	DW-MRI	DCE-MRI
Acquisition/sequence type	PET listmode, reconstruction with 2D-OSEM (4i16s)	2D RARE	Diffusion-weighted SE	2D FLASH
TE/TR [ms]	–	38/5841	42/1100	1.5/72
Voxel size* [mm^3^]	0.65 × 0.65 × 0.8	0.14 × 0.14 × 0.4	0.3 × 0.3 × 1	0.4 × 0.4 × 1.5
Slice gap (mm)	–	–	0.25	0.5
Image matrix	128 × 128	256 × 256	96 × 76	75 × 60
Flip angle (degree)		90	90	12
Contrast agent				Gadovist®
Injected activity [MBq]	12.1 ± 2.1	–	–	–
*b*-values [s/mm^2^]	–	–	0, 100, 200, 300, 400, 500, 600, 700, 800	–

*Including the slice gap

Irradiation with ten fractions of 2 Gy per day was applied for 2 weeks using a dedicated small animal image-guided RT platform (SAIGRT, Dresden, Germany) [36]. For irradiation, the animals were immobilized using plastic tubes fixated on a precisely movable carbon table; the tumor-bearing leg was positioned using a foot holder. Positioning accuracy with respect to the radiation field was checked with portal X-ray imaging (80 kV, 0.8 mA). All irradiations were performed using iso-centric opposed fields with dedicated circular collimators (8–14 mm diameter) depending on tumor volume. Radiation dose and corresponding irradiation time were calculated as a function of tumor size.

### ML-based identification of radioresistant clusters

#### Image pre-processing

During a data preprocessing step, the tumor region as well as a representative muscle region were defined manually based on the T2w-MRI data by an experienced radiation oncologist (SB) using the open-source software 3DSlicer. The tumor region was manually contoured on all image slices to encompass the whole lesion, excluding skin and bony structures. Resulting tumor volumes are summarized in Table 1. Muscle tissue was carefully contoured in the ipsilateral leg excluding bones and blood vessels. All quantitative MRI data were resampled to the PET image grid for subsequent processing and analysis. To correct for potential movements of the animal between different acquisitions, local rigid registrations between the respective images were performed using the open-source toolkit elastix (details on registration parameters are given in Supplementary Table S1). The registration result was carefully visually checked by an imaging scientist (SL) and a radiation oncologist (SB) and manually adjusted if necessary.

#### Extraction of quantitative parameter maps

Maps of apparent diffusion coefficient (ADC) values were derived from DW-MR images using a mono-exponential fit over all *b*-values with in-house software developed in python (scipy 0.19.1).

**Fig. 2:**
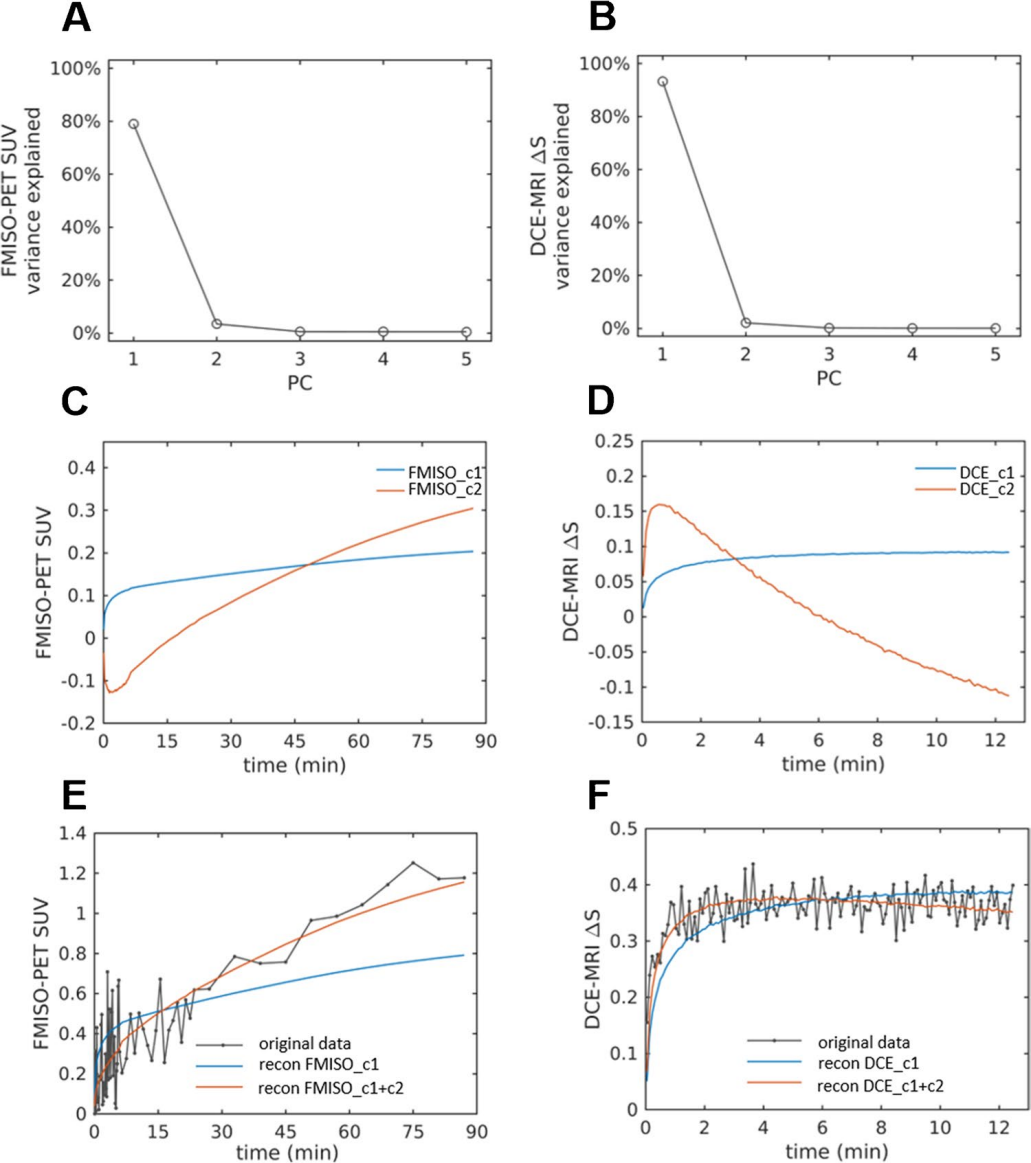
Principal component analysis (PCA) of dynamic imaging data for FMISO PET (**A**) and DCE-MRI (**B**). Upper row: Variance of data explained by first five principal components (PC). Middle: Time-dependent curves of principal components 1 and 2. Lower row: Exemplary image voxel with raw data of FMISO PET and DCE-MRI and time curve reconstructed by PC 1 only or PCs 1 and 2

FMISO PET data was first transformed into static uptake parameter maps by generating a tumor-to-muscle ratio map from normalized voxel activity concentration with respect to mean muscle uptake in the second last FMISO PET frame (approx. 80 min pi) to avoid potential artifacts caused by the following MRI contrast agent injection. To further extract quantitative parameter maps related to tumor hypoxia from dynamic FMISO PET signals, FMISO activity concentrations were converted into maps of standardized uptake value (SUV) by normalization to body weight and injected activity. Then, a principal component analysis (PCA) was performed using the uncentered data to extract a reduced set of quantitative parameter maps. Based on the variance explained by the individual principal components (PCs), the projection coefficients of the first two PCs (FMISO_c1, FMISO_c2) were found to be sufficient to describe the measured tracer dynamics and kept for further analyses (Fig. 2).

Similarly, for DCE-MRI, measured signal intensities ${S}_{{t}_{i}}$ were converted to relative signal increase

$$\Delta {S}_{{t}_{i}}=\frac{{S}_{{t}_{i}}-{S}_{0}}{{S}_{0}}$$

with ${t}_{i}=\left\{1, \cdots , 150\right\}$ being the time frames, and ${S}_{0}$ the baseline signal intensity, averaged over $11$ frames acquired prior to contrast agent injection. Quantitative parameter maps were then derived from $\Delta S$ data using PCA, yielding two final parameter maps containing the two first PC projection coefficients DCE_c1 and DCE_c2 (Fig. 2).

#### Model training for identification of radioresistant clusters

We propose a novel method for unbiased identification of tumor clusters defining HRS from multi-parametric quantitative imaging. This method is based on the hypotheses that recurrence after RT originates from such HRS inside the macroscopic tumor, which fails to be controlled by a standard radiation dose and fractionation due to its biological and physiological properties, and that a larger HRS translates into higher levels of radiation resistance. We therefore implemented a method which automatically extracts tumor clusters with similar biological and physiological properties as derived by joint information of quantitative maps from functional imaging and scores their ability to stratify tumor cell lines according to radiation sensitivity. In this way, relevant image parameters were learned which fulfill the hypotheses listed above.

**Fig. 3:**
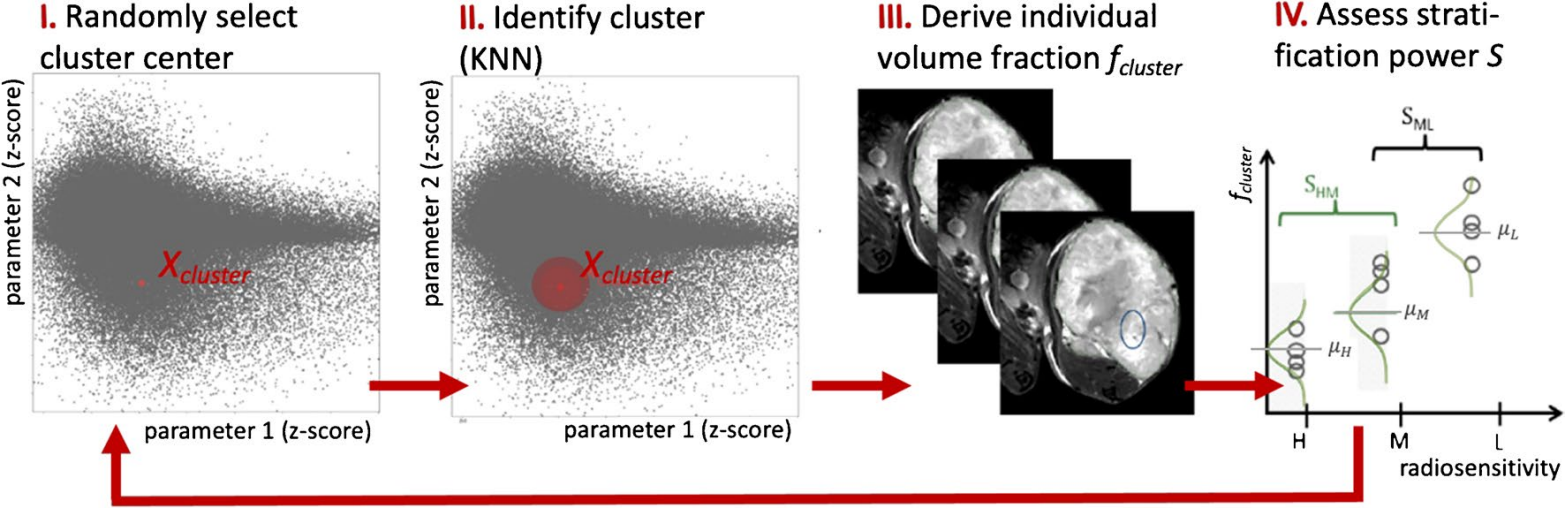
Preclinical model development. Schematical representation of machine learning model to identify clusters in multi-dimensional imaging space linked to radiation sensitivity: (I) Randomly select a cluster center in n-dimensional imaging parameter space. Each point in this 2D parameter plot corresponds to the corresponding parameter values of one tumor voxel in the cohort. (II) Identify the corresponding cluster using the K-nearest neighbor (KNN) clustering method. (III) Derive the fractional volume corresponding to this cluster in individual xenografts. (IV) Assess stratification potential S with respect to radiation resistance groups using Cohen’s *d*-score

A schematic overview of the machine learning approach to identify most relevant parameters in *n*-dimensional imaging space is provided in Fig. 3. For this analysis, only the imaging data cohort ${C}_{all}=42$, where all five quantitative parameter maps (ADC, FMISO_c1, FMISO_c2, DCE_c1, DCE_c2) were available for the first imaging time point, were included into the analysis (cf. Table 1). First, the total number of tumor voxels of the training cohort *C*_*all*_ was collected in common parameter spaces. 1- to 5-dimensional (1D to 5D) image parameter spaces were built, with each dimension being spanned by one of the five quantitative parameters extracted from functional imaging. Samples in parameter space (tumor voxels) were $z$-normalized. During parameter space scanning, each 1D to 5D parameter space was scanned for connected clusters of a fixed number *N*_*HRS*_ of voxels with similar parameters. According to [33], *N*_*HRS*_ was chosen such that the fraction of tumor voxels belonging to HRS resulted in 15.0%, 7.5%, and 0% for tumor cell lines of low, medium, and high radiation sensitivity, respectively.

Parameter space scanning was performed by repeating the following steps *N*_*it*_ = 5000 times: (1) randomly select one sample as cluster center *X*_*cluster*_; (2) assign its ${N}_{HRS}$ nearest neighbors (KNN clustering) using the Euclidean distance from *X*_*cluster*_ in parameter space as proximity measure; (3) derive the fraction of voxels in this cluster *f*_*cluster*_ for each individual tumor; (4) quantify the stratification potential of *f*_*cluster*_ using a stratification score *S*.

#### Quantification of stratification potential

For a robust, score-based assessment of the stratification potential for each tested parameter combination, cell lines were grouped into classes of distinct radiation sensitivity based on previously published tumor control doses (TCD50, Table 1) [32, 33]. Cell lines with overlapping confidence intervals were considered not distinguishable with respect to radiosensitivity and were therefore grouped into the same class. By doing so, three distinct classes of cellular radiation sensitivity could be identified: a class of high (H) sensitivity (UTSCC-45, XF354, UTSCC-14, UTSCC-8), medium (M) sensitivity (FaDu), and low (L) sensitivity (UTSCC-5, SAS). UTSCC-5 could not be successfully implanted into animals. Imaging data of the cell line CAL-33 could not be reproducibly analyzed due to significant differences in image quality; further, no reliable assignment of radiosensitivity class based on the high reported range of TCD50 was possible. Therefore, CAL-33 was excluded from the analysis.

The stratification potential, i.e., the capability to separate groups H-M and M-L, respectively, for any investigated parameter combination was quantified by Cohen’s *d* as effect size measure

$$S_{ij}=\frac{\;\mu_j-\mu_i}{\sigma_{ij}}\text{with}\left(i,j\right)\in\left\{\left(H,M\right);\left(M,L\right)\right\}.$$

Here, ${\mu }_{i,j}$ is the mean of the assessed HRS of group $i$ or $j$ based on the different parameter combinations, whereas ${\sigma }_{ij}$ is the pooled standard deviation of groups $i$ and $j$, defined as

$$\sigma_{ij}=\sqrt{\frac{\left(n_i-1\right){\cdot\;\sigma}_i^2+\left(n_j-1\right)\cdot\sigma_j^2}{\left(n_i+n_j-2\right)}}$$

with ${\sigma }_{i,j}$ being the group variances and ${n}_{i,j}$ the number of observations in groups $i$ or $j$, respectively. The final score was defined as the arithmetic mean

$$S=\frac{{S}_{HM}+{S}_{ML}}{2} .$$

#### Selection of optimal HRS clusters in 1D to 5D imaging space

For each *n*-dimensional image parameter space, the clusters yielding the highest stratification score ${S}_{HRS, nD}$ and their corresponding cluster centers *X*_*HRS,nD*_ were identified and used for comparing the performance of different parameter spaces. Furthermore, the differences of ${f}_{HRS, nD}$ between radiosensitivity groups H-M and M-L, respectively, were tested for significance using a Wilcoxon rank sum test. *P* < 0.05 was considered statistically significant.

#### Assessment of robustness

To evaluate the robustness of the identified stratification scores ${S}_{HRS}$ and their cluster centers *X*_*HRS*_, an internal bootstrap validation was performed for each parameter space. Each bootstrap cohort was drawn with replacement from the original training cohort *C*_*all*_, using a total number of *N*_*bs*_ = 50 bootstrap cohorts. Robustness was then quantified by deriving bootstrap-based 95% confidence intervals (CIs) for ${S}_{HRS}$ and *X*_*HRS*_, respectively. For an additional assessment of the robustness of *X*_*HRS*_, the distribution of identified scores after parameter space scanning was visualized as multiple 2D projections.

#### Extended cohort

To test the resulting ML models for the best models identified during training, model verification was performed using an extended cohort. For this purpose, an extended cohort consisting of all animal data available for the respective parameter combination *C*_*max*_ was used including also incomplete data sets not used during training (cf. # imaging data sets given in brackets in Table 1).

### Classical imaging parameters and multiple time points

For comparison, classical ADC-related imaging parameters reporting mean, minimum, and maximum value in a tumor were reported. In addition, *ADC*_*valley*_ was derived by the minimum ADC value in a connected image region of seven voxels to create a robust measure related to minimum ADC but unaffected by artifacts originating from partial volume effects at the edges of the tumor. Similarly, maximum and peak values of FMISO tumor-to-muscle ratio (${TMR}_{max/peak}$) and mean, maximum, and peak as average over seven voxels around the maximum FMISO SUV were calculated using the late PET frame acquired 80 min p.i. for each tumor and correlated to cell-line specific radio sensitivities. The full analysis pipeline described above was also carried out for imaging data acquired after 2 weeks of fractionated RT (w2).

## Results

**Fig. 4:**
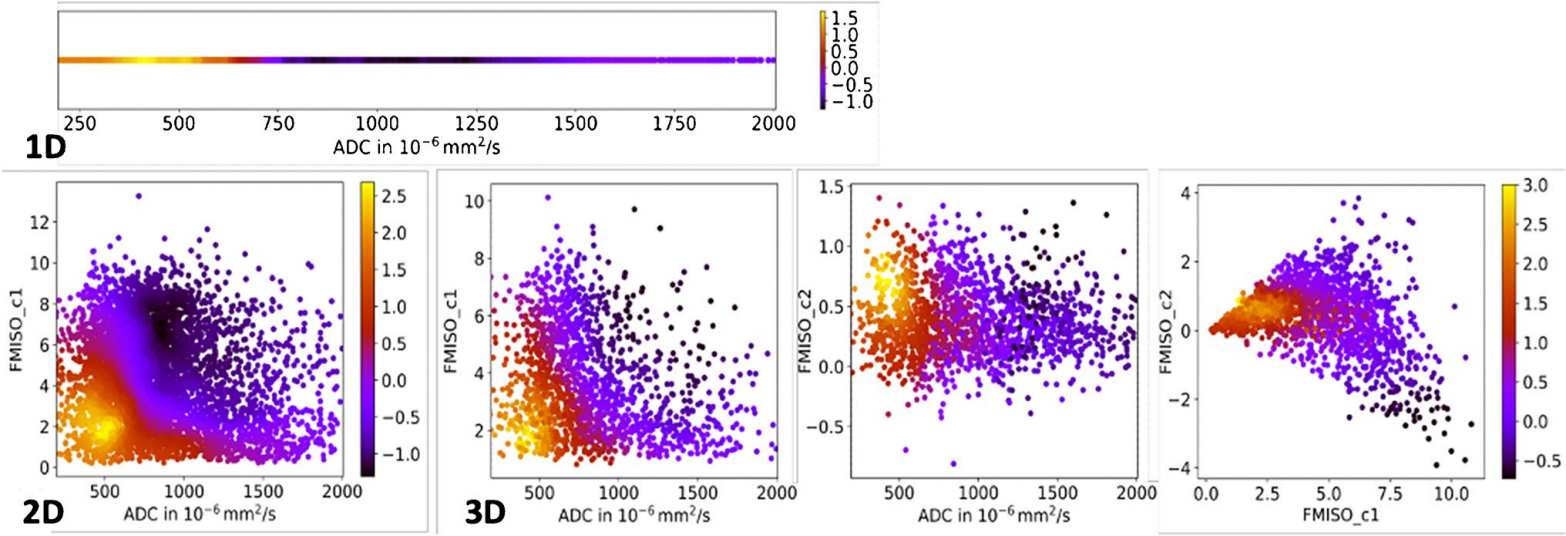
Visualization of stratification scores in 1D to 3D parameter space. Stratification scores S for the best-scoring 1D, 2D, and 3D imaging parameter spaces. 3D parameter space is shown as corresponding 2D projections for better visualization

**Fig. 5:**
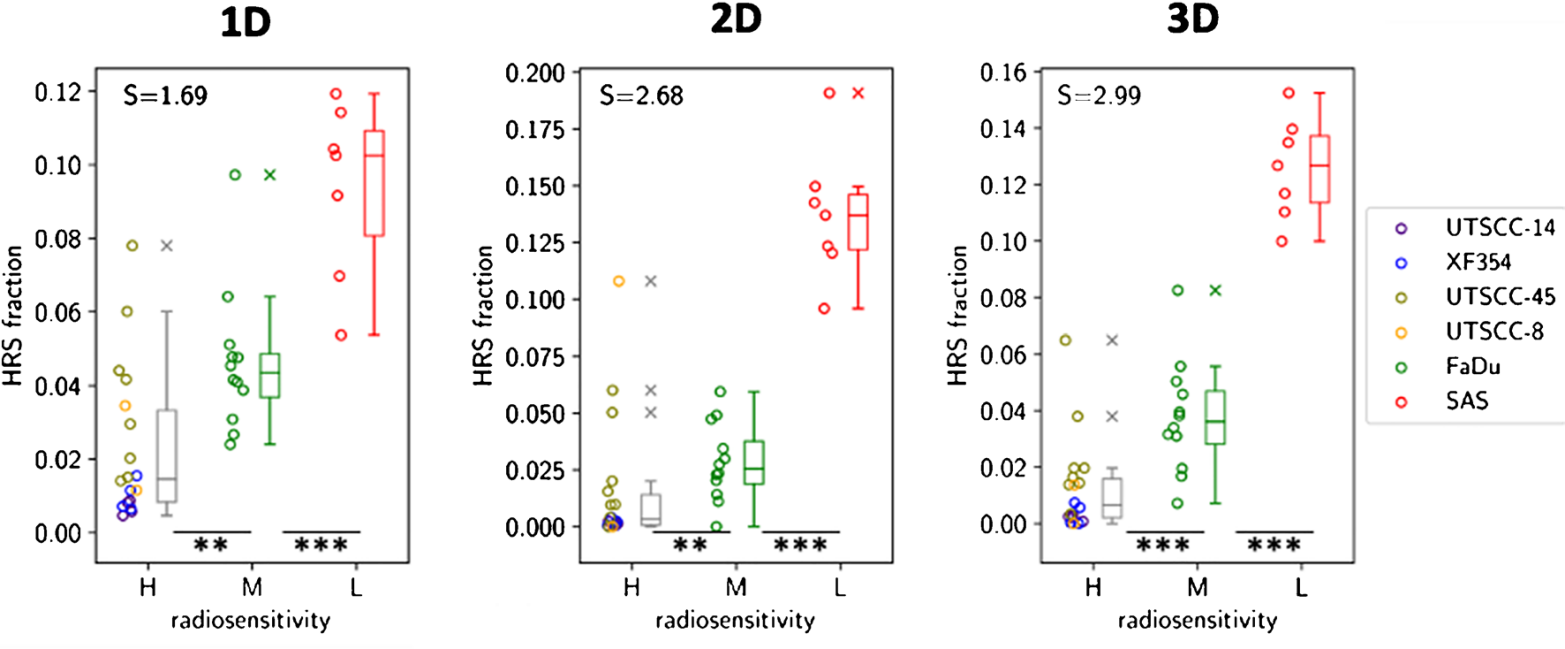
Stratification potential of 1D to 3D clusters. Box plots showing significant stratification for data cohort ${C}_{all}$ of best scoring 1D, 2D, and 3D imaging clusters according to high (H), medium (M), and low (L) radiation resistance including Cohen’s *d*-score S for each cluster. HRS fraction is defined by the relative HRS of each sample normalized to the whole tumor volume

**Fig. 6:**
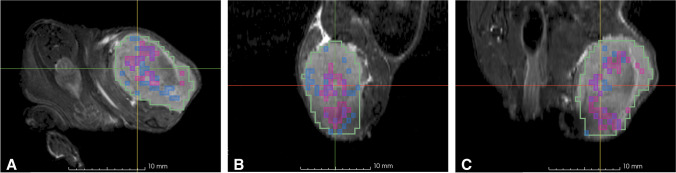
Visualization of 1D and 3D HRS clusters in addition to ADC_valley_. Example of 1D (blue) and 3D (purple) HRS annotations inside a SAS-tumor, in axial (**A**), sagittal (**B**), and coronal views (**C**) overlaid to the anatomical T2w-MRI. The position of the crosshair defines the center of the region with lowest ADC_valley_. Gross tumor volume (GTV) delineation is shown in green. Voxel structure of contours results from resampling of all functional data and GTV delineation to the PET image grid, which had the lowest resolution

During model training in 1D to 5D search space on the baseline imaging data, we identified distinct clusters in 1D to 3D imaging parameter space which were able to significantly stratify the xenograft tumors according to their radiation resistance. Using 1D parameters only, the best stratifying cluster was obtained for ADC derived from DW-MRI which showed the highest stratification power with an effect size [95% CI] of ${\mathrm{S}}_{\mathrm{HRS},1\mathrm{D}}=1.69 \left[1.46-3.20\right]$, $p=0.002$. Interestingly, the center (interval) of the cluster was located at ${\mathrm X}_{1\mathrm D,\mathrm{ADC}}=420\left[384;457\right]\cdot10^{-6}\text{mm}^2\text{/s}$, which corresponded to the left flank of the histogram generated from all animals. In comparison, we found significantly increased stratification potential for a 2D cluster defined by ADC and FMISO_c1 (${\mathrm{S}}_{\mathrm{HRS},2\mathrm{D}}=2.68 \left[2.41-4.12\right]$, $p=0.01$). Details on cluster center and interval in terms of imaging parameter values are given in Table 3. The best stratifying cluster in *n*-dimensional imaging space was spanned by the 3D quantitative maps of ADC, FMISO_c1, and FMISO_c2, yielding an effect size of ${\mathrm{S}}_{\mathrm{HRS},3\mathrm{D}}=2.99 \left[2.50-4.44\right]$, with $p<0.0001$ respectively. When further increasing the dimensionality of the parameter space, further improvement of ${\mathrm{S}}_{\mathrm{HRS}}$ was observed, which, however, was not significant ($p>0.05$) according to Mann-Whitney *U* test based on a bootstrap analysis with respect to ${\mathrm{S}}_{\mathrm{HRS},3\mathrm{D}}$. Best scoring models in 1D to 5D imaging space are summarized in detail in Table 3. A visualization of the *n*-dimensional search space is presented in Fig. 4, whereas Fig. 5 shows the corresponding stratification potential for the selected 1D, 2D, and 3D clusters. Figure 6 presents an example of one preclinical tumor (SAS) with annotations of 1D and 3D HRS.

**Table 3:** Best scoring parameter combinations. Stratification potential of multi-dimensional imaging clusters at baseline before RT for all 1D to 5D combinations. Best stratifying combinations are printed in bold

	Parameter	Stratification Score *S* [95% CI]	Cluster center^a^ [interval]	*p*-value
1D	ADC	**1.69 [1.46; 3.20]**	**420 [384; 457]**	**0.002**
1D	FMISO_c1	1.24 [1.02; 2.17]	1.26 [1.07; 1.45]	0.07
2D	ADC FMISO_c1	**2.68 [2.41; 4.12]**	**492 [358; 627]** **1.64 [0.48; 2.81]**	**0.01**
2D	ADC FMISO_c2	2.40 [2.09; 3.67]	434 [307; 564] 0.12; 0.74]	< 0.0001
3D	ADC FMISO_c1 FMISO_c2	**2.99 [2.50; 4.44]**	**439 [230;652]** **1.81 [0.61; 3.66]** **0.71 [0.19; 1.21]**	** < 0.0001**
3D	ADC FMISO_c1 DCE_c1	2.99 [2.63; 4.31]	478 [303; 658] 1.79 [0.36; 3.35] 0.51 [− 0.33; 1.34]	0.022
4D	ADC FMISO_c1 FMISO_c2 DCE_c2	3.17 [2.77; 4.85]	487 [183; 796] 2.75 [0.55; 5.36] 0.63 [− 0.10; 1.34] 0.10 [− 0.14; 0.34]	0.001
4D	ADC FMISO_c1 FMISO_c2 DCE_c1	3.15 [2.60; 4.60]	439 [204 [694] 1.81 [0.48; 3.97] 0.71 [0.10; 1.32] − 0.05 [− 0.87; 1.14]	0.005
5D	ADC FMISO_c1 FMISO_c2 DCE_c1 DCE_c2	2.79 [2.51; 4.73]	385 [93; 695] 2.12 [0.37; 4.80] 0.56 [− 0.14; 1.27] 0.43 [− 0.87; 1.80] − 0.16 [− 0.40; 0.09]	0.006

^a^Cluster centers and intervals are given in [mm^2^/s] for ADC and [] for FMISO_c1, FMISO_c2, DCE_c1 and DCE_c2

**Fig. 7:**
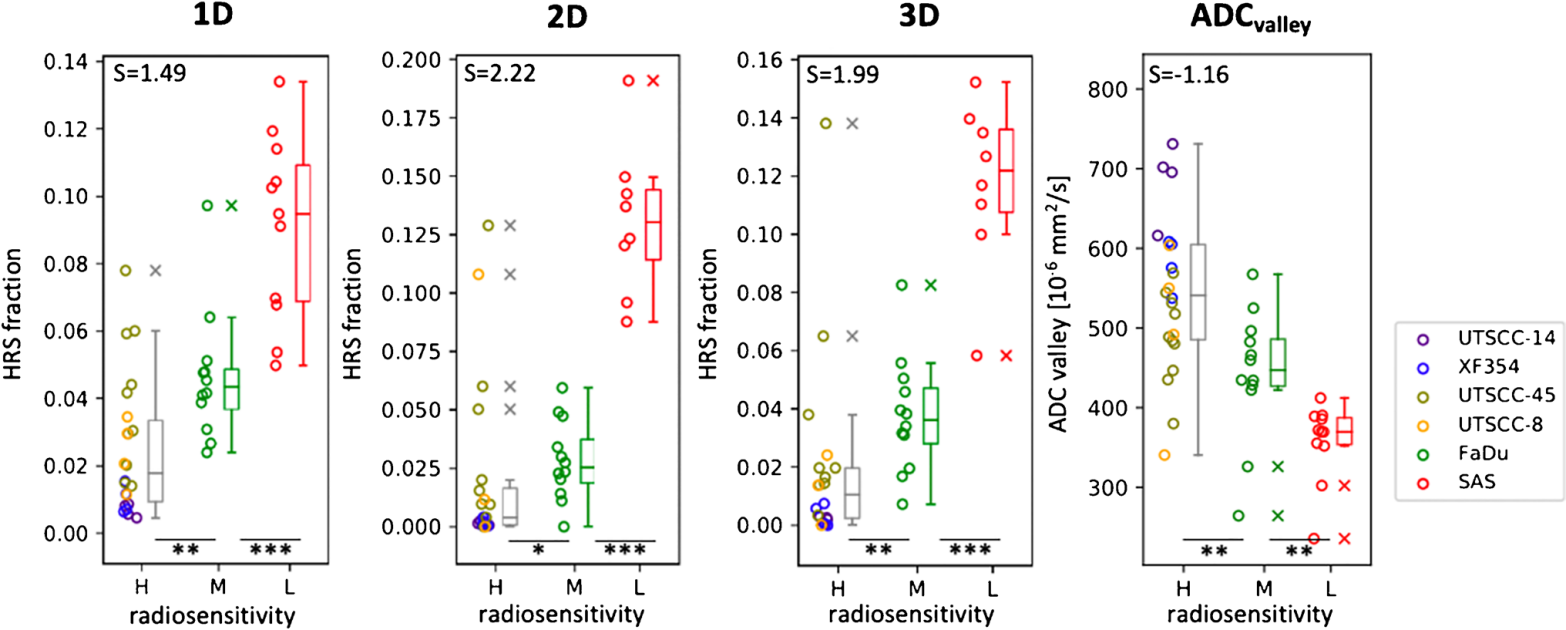
Verification of stratification potential for extended cohorts ${C}_{max}$ of 1D to 3D clusters and ADC_valley_. Box plots showing significant stratification for data of extended cohorts ${C}_{max}$ of best scoring 1D (*N* = 51), 2D (*N* = 45), and 3D (*N* = 45) imaging clusters according to high (H), medium (M), and low (L) radiation resistance in comparison to ADC_valley_ (*N* = 51) including Cohen’s *d*-score *S* for each cluster. HRS fraction is defined by the relative HRS of each sample normalized to the whole tumor volume

Correlation of cell line specific radiation sensitivities with the classical imaging parameters in the tumor region did only yield significant stratification potential for ${ADC}_{valley}$ ($p=0.006$), cf. Figure 7 and Table 4.

**Table 4:** Classical imaging parameters. Stratification potential of imaging parameters at baseline before RT related to FMSIO TMR and SUV values as well as ADC. Peak and valley parameters are calculated by an average value of seven voxels centered around the maximum or minimum value in the tumor, respectively

Parameter	Stratification score *S*	*p*-value
FMISO TMR_peak_	− 0.12	0.256
FMISO TMR_max_	0.02	0.367
FMISO SUV_peak_	− 0.17	0.160
FMISO SUV_max_	0.00	0.217
FMISO SUV_mean_	− 0.48	0.082
ADC_max_	− 0.29	0.416
ADC_mean_	− 1.26	0.122
ADC_min_	− 0.57	0.095
ADC_valley_	− 1.22	**0.006**

Significant results (*p *< 0.05) are printed in bold

Figure 7 shows the validation results for the best 1D, 2D, and 3D models identified during training in addition to the only significant classical parameter ${ADC}_{valley}$. Stratification results of the different models in the extended cohorts ${C}_{max}$ are similar to those obtained the training cohort ${C}_{all}$, indicating high robustness of the method.

Following the same methodology, 1D to 5D parameter space scanning was performed for imaging data obtained after 2 weeks of fractionated RT. Here, only a 1D cluster defined by the FMISO_c1 map measured in w2 yielded significant stratification potential ${\mathrm{S}}_{\mathrm{HRS},1\mathrm{D},\mathrm{ w}2}=1.12 \left[0.90-3.69\right]$, $p=0.041$. Results of *n*-dimensional model training in w2 of RT are detailed in Table 5.

**Table 5:** Results of ML cluster analysis after two weeks of radiotherapy. Best stratifying multi-dimensional imaging clusters after two weeks (w2) of fractionated RT. Best stratifying combinations are printed in bold

	Parameter	Stratification score *S* [95% CI]	Cluster center^a^ [interval]	*p*-value
1D	FMISO_c2	1.21 [0.87; 1.66]	1.07 [0.96; 1.18]	0.402
1D	FMISO_c1	**1.12 [0.90; 3.69]**	**0.70 [0.56; 0.83]**	**0.041**
2D	ADC FMISO_c1	2.94 [2.55; 5.31]	752 [612; 892] 1.09 [0.09; 2.09]	0.430
2D	ADC DCE_c1	2.40 [1.93; 4.06]	704 [533; 875] − 0.37 [− 1.07; 0.33]	0.094
3D	ADC FMISO_c1 DCE_c1	2.57 [2.44; 5.36]	811 [650; 972] 1.13 [0.07; 2.27] 0.05 [− 0.59; 0.71]	0.402
3D	ADC FMISO_c1 FMISO_c2	2.56 [2.38; 4.14]	771 [554; 990] 0.68 [0.05; 2.22] 0.24 [− 0.03; 0.50]	0.207
4D	ADC FMISO_c1 FMISO_c2 DCE_c2	2.70 [2.43; 5.57]	644 [149; 1193] 1.48 [0.66; 5.47] 1.08 [0.39; 1.70] 0.49 [− 0.77; − 0.17]	0.375
4D	ADC FMISO_c1 FMISO_c2 DCE_c1	2.47 [2.26; 4.81]	758 [520; 994] 0.68 [0.05; 2.34] 0.25 [− 0.04; 0.53] 0.23 [− 0.71; 1.09]	0.178
5D	ADC FMISO_c1 FMISO_c2 DCE_c1 DCE_c2	2.40 [2.07; 4.74]	460 [18; 940] 0.63 [0.05; 3.77] 0.21 [− 0.23; 0.78] 0.25 [− 1.20; 1.91] 0.12 [− 0.15; 0.33]	0.486

^a^Cluster centers and intervals are given in [mm^2^/s] for ADC and [] for FMISO_c1, FMISO_c2, DCE_c1 and DCE_c2.

## Discussion

In this study, we report pre-clinical training of a multi-dimensional PET/MRI-based QIB to detect HRS in HNC as potential target for future focal dose escalation. Our findings suggest that a HRS defined by a cluster of ADC values derived from DW-MRI correlates spatial maps of cellularity with individual radiation resistance considering a 1D quantitative functional imaging map as input. Highest stratification potential with respect to cell line specific radiation resistance was found for a 3D QIB created from ADC, and two PCs of dynamic FMISO PET information. Increasing dimensionality further did not significantly increase stratification potential, which may be due to redundancies hidden in the n-dimensional functional imaging data. Consequently, we identified a QIB profile from PET/MRI using a novel machine learning approach in a pre-clinical setting. Starting from a wide search approach with as few assumptions as possible using the main quantitative imaging techniques which are clinically available today, we were able to identify the most promising multi-parametric QIB for potential usage for future RT individualization.

The proposed method relies on the identification of a radioresistant cluster in parameter space only. Consequently, we do not per se assume a spatially connected area of the HRS inside the tumor. If spatial connection is given, HRS may be used for potential future local radiotherapy interventions, such as dose painting. If HRS voxels in contrast would be scattered throughout the tumor, this might be indicative of a generally more radioresistant tumor and dose painting strategies may result in a radiation dose escalation of the whole tumor. However, scattered HRS voxels throughout the GTV might also be caused by noise and potentially weak robustness of the model, which should be clarified in future validation studies in preclinical and ultimately also clinical settings.

Due to their limited size and heterogeneity, direct application of the ML models to identify spatially connected HRS regions in patients may not be possible. In this study, eight different cell lines with distinct radiation resistance levels were used, meaning that each small animal tumor must be understood as a role model for one voxel of a patient tumor. Consequently, the final model may not necessarily yield connected HRS areas but will require retraining and validation in patients.

ADC has been identified by earlier studies as potential prognostic QIB in HNC [8, 16], whereas other studies reported controversial results [37]. The discrepancy of earlier results may be due to over-simplified imaging measures such as mean ADC averaged over the whole tumor in contrast to the sub-volume approach based on clusters in multi-dimensional QIB space proposed in this study. Classical or global imaging parameters investigated in this study demonstrated that ${ADC}_{valley}$ appears to also be associated with radiation sensitivity. A potential explanation for this observation might be that ${ADC}_{valley}$ is a mean value calculated from seven voxels around the minimum $ADC$ in a tumor sample and may thus be correlated to the 1D cluster identified during ML training on voxel level.

However, when using joint information from ADC maps derived from DW-MRI combined with two PC of dynamic FMISO PET, significantly better stratification was obtained compared to ADC only. This comes however to the expense of acquiring in addition to DW-MRI dynamic hypoxia PET which increases the level of complexity during patient examination and image acquisition enormously. So far, only small hypoxia PET patient data sets were reported due to the complexity of acquisition requiring experimental tracer production, extended scan times, and non-standard data analysis strategies which make a broad roll-out of this technology unrealistic [5]. Nevertheless, these findings corroborate earlier results reported by our group and others that dynamic hypoxia PET has prognostic character with respect to RCT outcome [7, 12, 15]. Assuming that repeated functional imaging will further enhance the power of image-based adaptive RT interventions, it appears that dynamic hypoxia PET is more complex, costly, and not as broadly available as DW-MRI. Thus, from a pragmatic point of view, DW-MRI appears promising for wider clinical roll-out with change of practice even if less predictive than 3D-HRS combining DW-MRI and FMISO PET.

Analysis of the preclinical imaging data acquired 2 weeks after fractionated RT revealed no stratification of radiation resistance groups for most cluster combinations. Sole hypoxia PET yielded slightly significant stratification power at this time point early during RT. As such, this confirms clinical findings of prognostic potential of FMISO PET at the second week during RT [14, 38]. However, in this study, the model for w2 was newly trained without any inference from the models obtained for pre-treatment data.

Our ML approach used to identify multi-dimensional clusters of radiation resistance is based on several assumptions. First, radiation resistance levels were based on data from earlier pre-clinical studies [32, 33], showing significant variation in radiation resistance between experiments. Second, small animal functional imaging is extremely challenging, requires anesthetized animals, and thus deviates from a standard clinical situation. In addition, we assumed a relative HRS size varying between 0 and 20% depending on the radio-resistance levels of the respective cell lines. A further drawback of our method is the fact that parameter space scanning was performed directly on image voxel data, which is more prone to noise and registration inaccuracies compared to volume averaged methods. An alternative would be to combine single voxels to small homogeneous subregions (supervoxels) prior to parameter space scanning, e.g., by means of simple linear iterative clustering [19].

In this study, we used a data-driven ML approach in terms of PCA for extracting a reduced number of QIB maps from dynamic functional imaging. The use of PCA for dynamic data has been shown to be promising by other clinical and pre-clinical studies [39] providing potentially more robust results compared to classical use of compartment models for such data [7, 9].

A previous study proposed deriving high-risk tumor subvolumes from joined functional imaging information by clustering patient imaging data [19]. However, this method does not directly use the size of a HRS for patient stratification but apply different intermediate steps to determine heuristic stratification parameters. In contrast, our method uses the relative HRS size which is directly connected to cell line specific hypoxia levels which are only available in a translational approach. This prior represents a major limitation of our study, as no tumor specific hypoxia or radiation resistance levels were measured. This underlines the necessity of independent validation studies, ideally in patients to confirm the hypotheses identified in this experiment.

Potential uncertainties of the method making use of multi-dimensional functional imaging data on voxel level originate from manual contouring of tumor regions used as input for the analysis as well as co-registration of the functional imaging data sets which is of crucial importance for the integrity of the data set in higher dimensions. Robustness of the proposed HRS method was evaluated in different ways. The density of visualized scores in parameter space (Fig. 4) shows a smooth distribution as well as a single, compact region of high scores ${S}_{HRS}$, indicating robust learning of the cluster center ${X}_{HRS}$, which is further supported by the internal bootstrap validation using the training cohort ${C}_{all}$. Furthermore, robustness of the model was evaluated using an extended cohort ${C}_{max}$ including additional tumors which were not part of the initial training cohort ${C}_{all}$. Even though this evaluation indicated stability of the model parameters, this approach cannot be considered a full independent validation due to only a small number of additional data sets in ${C}_{max}$ compared to ${C}_{all}$. A potential alternative for tumor stratification based on joint QIB maps might be an end-to-end learning approach using for example convolutional neural networks (CNNs), which have shown to achieve high performance in image processing and classification tasks [40]. We did not investigate such approach since we had only a low number of tumors with the full multi-dimensional imaging parameter space available in this study (*n* = 42). Therefore, an approach was developed which complements a data-driven learning method with hypotheses about the existence and size of an HRS related to known radio-resistance levels. The final model can easily be interpreted in the sense that learned HRS are fully determined by associated QIB ranges. In contrast, model interpretation using CNN-based end-to-end learning might be challenging.

In Fig. 5, cell line UTSCC-45 shows distinctly different HRS compared to all other cell lines of the group with high radiation sensitivity (group H). Interestingly, this cell line differs from the other investigated cell lines due to its positive human papilloma virus (HPV) status. The associated genetic difference may cause a shift in radiosensitivity compared to HPV-negative cancer cell lines which seems not to be detectable by quantitative imaging [41]. Therefore, ADC/FMISO-based HRS radiation dose escalation does not seem an option for low-risk HPV-positive oropharyngeal HNC and future interventional trials should be limited to patients with high-risk profiles (HPV-negative or HPV-positive plus  > 20 pack-years smoking history) [42].

As tumor hypoxia and cellularity are subject to change during RCT, individualized RT approaches adapted to the current level of resistance will only be possible if HRS can be identified shortly before treatment. Recently developed hybrid MR-Linacs may allow functional MRI acquisitions before and during RT and open thus unique possibilities in terms of MR-specific QIB-adaptive RT [43]. Recent results on phantom and early clinical data proved that quantitative imaging is possible at hybrid MR-Linac systems [44, 45] which is a major pre-requisite for biologically adapted RT dose painting based on ADC clusters. More complex multi-parametric QIB involving different imaging modalities may need to be acquired on dedicated PET/MRI scanners and used for offline response-adaptive RT. Nevertheless, before QIB-based RT dose painting can be applied in clinical RT practice, technical and clinical validation is required including test–retest studies and comparison to diagnostic scanners to ensure repeatability and reproducibility [43, 46].

In conclusion, this study used a novel ML approach combined with hypothesis-driven methods, where *n*-dimensional imaging spaces spanned by hypoxia imaging using dynamic FMISO PET, DW-MRI, and DCE-MRI were scanned to learn characteristic patterns of radiation resistance. Finally, we present the pre-clinical description of a HRS defined by a 3D cluster defined by ADC, FMISO_c1, and FMISO_c2 which identifies spatially resolved tumor subvolumes exhibiting increased radiation resistance and thereby presumably the cause of local tumor recurrence. These results warrant validation and translation to a clinical setting before benefits of PET/MRI-derived, QIB-based RT adaptation can be tested in a clinical trial.

## Supplementary Information

Below is the link to the electronic supplementary material.

Supplementary file1 (PDF 23 KB)

## Data Availability

The datasets generated during this study are available from the corresponding author on reasonable request through institutional data transfer agreements.
