# Supplementary material for: Machine learning identifies multi-parametric functional PET/MR imaging cluster to predict radiation resistance in preclinical head and neck cancer models
Source: Eur J Nucl Med Mol Imaging. 2023 May 6;50(10):3084–96. doi: 10.1007/s00259-023-06254-9 (PMC10382355; doi:10.1007/s00259-023-06254-9)
Supplement: Supplementary file 1 — Supplementary file1 (PDF 23 KB) [file 259_2023_6254_MOESM1_ESM.pdf]

Supplementary Table S1. Image registration parameters. Settings used for rigid registration with the open-source toolkit *elastix*.

Item	Parameter
Registration	Multi Resolution Registration
Number of Resolutions	3
Transform	Euler Transform
Metric	Advanced Mattes Mutual Information
Number of Histogram Bins	64
Optimizer	Adaptive Stochastic Gradient Descent
Number of Spatial Samples	5000
Image Sampler	Random
Maximum Number of Iterations	4000
